# Guy's Hospital Reports. Second Series

**Published:** 1847-01-01

**Authors:** 


### Guy's Hospital Reports. Second Series. Edited by Drs. *Barlow* and *Birkett*, and Messrs. *Cock* and *Poland*. Vol. IV. 8vo. pp. 498. Highley: London, 1846.

The present volume contains papers by Drs. Addison, Lever, Williams, and Hughes, and by Messrs. France, Hilton, King and A. Taylor; besides two Reports, one Medical and the other Surgical, of the Clinical Society connected with the Hospital. We shall notice them *seriatim*.

### I. On the Fallacies attending Physical Diagnosis in Diseases of the Chest. By *Thomas Addison*, M.D. &c.

After a glowing eulogium on the rare merits of Laennec and the distinguished services which he has conferred upon medicine, by the introduction of Auscultation into the art of diagnosis, Dr. Addison very judiciously remarks that this great discovery has, on the whole, been more injured by indiscreet and indiscriminate advocacy of its claims than by the most determined and open hostility.

"Books and essays without number, and of great value, have been written for the purpose of adding favourable testimony to the merits of the stethoscope; to increase its utility, and extend its application; whereas, so far as I know, not a single individual has deemed it right, or desirable, to pursue the opposite course, of expressly publishing to the world, the manifold difficulties and fallacies attending its use. The publications alluded to, by the semblance of a too exclusive advocacy, have, according to my humble belief, placed the stethoscope and its pretensions in a false position; they have awakened in the minds of many a vague notion of infallibility; they have led the profession and the public to expect too much; and, by suppressing or concealing the real imperfections of a favourite expedient, have put it in the power of hostile parties to inculpate the stethoscope for the errors of the stethoscopist." P. 3.

No sensible physician will ever *substitute* Auscultation for other means of diagnosis; but he will gladly employ it as a most useful auxiliary. He will not lean upon it as a crutch, but only use it as a staff with which to explore his way. Never will he omit to obtain a full and complete history of every case of disease which comes under his care. It is not the knowledge of one symptom, or of one set of symptoms alone, that he is to be satisfied with; and how can he hope for such information unless by putting into requisition every means in his power of interrogating Nature? The too exclusive stethoscopist will unquestionably be oftener at fault, at least in his treatment, than he who refuses to avail himself of physical diagnosis altogether; but surely the abuse of a thing can be no reason, in the eyes of a man of sense, for the total neglect of it. As a matter of course, the acquisition of any new method of investigation pre-supposes an expenditure of diligent attention on the part of the student. Dr. Addison says: "unfortunately, in the medical profession, all truly valuable and practical knowledge, is only to be obtained by a proportionate sacrifice of time and labour."—Why *unfortunately?* It would be much better, alike for the public benefit and the credit of our profession, if the art of medicine—we do not say, of mere prescribing,—were not so easily attained. Dr. A. has cast his observations into the form of distinct propositions, appending remarks to each of them. We shall select those which strike us as the most important.

1. Many persons, whilst under examination, fail to perform the respiratory act efficiently, either from mere nervousness, or from not knowing how to do it; they merely heave the chest up and down, instead of freely inhaling and expelling the air.

This may lead to the erroneous belief that the respiratory murmur is deficient or even absent, while the lungs are perfectly healthy. The best way of preventing this source of fallacy is to desire the person to cough, and instantly afterwards to inspire forcibly, in order to cough a second time. The two sides of the chest should also be examined and compared together at the same time, in order to guard against error.

2. Whatever lessens the freedom, mobility, or elasticity of the ribs, renders the sound on percussion more dull.

Hence, in rickets, old age, &c. the signs afforded by percussion, and in many instances by auscultation also, are often little to be relied upon. The chest is imperfectly resonant, the respiratory murmur is apt to be unusually feeble, and the impulse of the heart to be much weaker than it would be, if the ribs were more free and elastic in their movements.

3. When there is lateral flattening of the ribs, with projection of the sternum, as is usually the case in a rickety patient, the action of the heart is liable from slight causes to beat with such violence, and to have its sounds and impulse so extensively diffused, as not unfrequently to have led to an unfounded apprehension of serious organic lesion of that organ.

The physician must, moreover, be more than usually cautious in his diagnosis and treatment of acute pulmonic disease under such circumstances; as the violence of the symptoms is often quite disproportionate to the extent and severity of the existing disease, and may therefore suggest unnecessarily active treatment.

4. When exploring the chest in a case of recent disease, we may be misled by the permanent effect of an ancient Pleurisy, or Pneumonia.

It is well known that an old pleurisy or pleuro-pneumonia will often cause very considerable contraction of the affected side of the thorax.

This deformity is generally observed at the lower part of the chest, either anteriorly or posteriorly, or both. As we might expect, there is usually dulness on percussion over the seat of a Pleuritic contraction, also feebleness or absence of respiratory murmur, constrained movements of the ribs, and dry crepitating or crackling sounds heard chiefly during the act of inspiration. In the same manner, there may be dulness on percussion, bronchial respiration, bronchophony, and a sub-mucous crepitation discoverable in a fever patient from an old attack of Pneumonia, which has left a larger, or smaller part of the lungs more or less completely hepatised. In either of these cases, it may be scarcely possible for the physician to determine, at least by mere physical examination, whether the consolidation be of recent or of ancient date.

As a matter of course, the existence of these signs might seriously mislead a physician in the diagnosis of recent disease, if he were not aware of their long standing. Hence the necessity of making minute enquiries as to the past history of a patient.

5. Distension of the abdomen from Ascites or from enlargement of the Liver or Spleen, &c. in short whatever tends to push up the diaphragm and to prevent the free expansion of the lungs, may have the effect of producing dulness upon percussion and feebleness of the respiratory murmur in the inferior part of one or of both sides of the chest; and may thus give rise to the suspicion of a Pleuritic Effusion being present, when all is right within the thorax.

The position of the patient, according as this is upright or horizontal, will necessarily have the effect of modifying the extent and degree of these abnormal signs just mentioned.

6. Of all the sources of fallacy to be encountered in the physical diagnosis of diseases of the lungs, Bronchitis is by far the most prolific of mistakes and oversights.

The co-existence of this disease is exceedingly apt to obscure the auscultatory symptoms of Phthisis, Pneumonia, and Pleurisy, as well as various forms of chronic pulmonary disease.

The value of the following remarks, on the complication of bronchitis with tubercular disease of the lungs in its early stage, will be appreciated by every practical physician :—

"It is under such circumstances that the too exclusive stethoscopist is liable to be beaten in diagnosis by those who reject physical examination altogether; for, the latter inquires carefully into the history of the patient and of his family, he observes attentively the patient's general aspect, and the character and order of the general symptoms; all of which are wont to be too much disregarded by the stethoscopist. Nevertheless, if the stethoscopist do his duty, he has greatly the advantage: he will institute an equally careful inquiry; added to which, he will observe whether the bronchial obstruction is limited to the apex of the lung; he will repeatedly, and for some minutes at a time, apply the stethoscope, or, what I prefer, the naked ear, to the upper part of the chest: he will desire the patient to breathe freely, to cough, and, if possible, to expectorate. He will, by so doing, often succeed in removing the obstructing mucus, and thereby develop, however slightly, some degree of bronchial respiration, or bronchophony, or both; signs strongly confirmatory of phthisical disease in doubtful cases. According to my own experience, the individual symptom, which, without being decisive, above all others increases the apprehension of phthisical disease, is occasional slight hæmoptysis just sufficient to tinge or streak the sputa." P. 9.

We are inclined to attach more importance than Dr. Addison seems to do to the character of the sputa. In simple Bronchitis we never observe those fragmentary boiled-rice-like particles floating in the water (in which the sputa have been received) and gradually settling down to the bottom of the vessel, which are almost uniformly seen in cases of genuine Phthisis. The streaked yellow appearance too of the expectorated matter, as well as the globular or nummulated form which it so often assumes, are phenomena nearly peculiar to the latter disease. Moreover, careful percussion will often serve most materially to aid the diagnosis. A firm or forcible tap over the site of a tubercular deposit very generally occasions, if not actual pain, a sense of uneasiness, which is not experienced by the patient at the corresponding spot on the opposite side of the chest; this uneasiness often extends through to the back of the shoulder.*

7. In every case of suspected Phthisis, the patient should be made to breathe and cough with some degree of violence, the physician having his ear applied all the while to the chest; as the bronchi opening into a tubercular cavity are apt to become occasionally obstructed with mucus, so that the characteristic auscultatory phenomena may not be perceived during gentle respiration. It is necessary, therefore, that the obstructing mucus be dislodged, before these phenomena can be distinctly recognised. From want of the simple precaution just given, many errors of diagnosis have been committed.

8. Dr. Addison remarks, with great truth, that "a person may have a violent tearing cough, lasting for weeks or months, attended with slight mucous expectoration, occasionally even streaked with blood, and causing pain to be felt throughout the whole chest; as well as an appearance of constitutional distress; whilst neither auscultation nor percussion can detect any morbid change in the chest."

Such a case may result from a very simple cause—one that is very often overlooked in practice—we mean, a relaxed Uvula. It may depend, in the female, on Hysterical irritation. We have seen it in several instances in persons of a Gouty diathesis, and found it yield to the use of antacids and of an anti-arthritic regimen, when it had resisted every other plan of medication. The existence of old pleuritic adhesions, or of limited pulmonic consolidation always, as might be expected, aggravates the severity and obstinacy of such coughs.

"9. When Pneumonia occurs in its simplest form, *i. e.* with little or no bronchial complication, there is sometimes no cough and consequently no expectoration;

---

* We may here remark, *en passant*, that the application of a single leech every night or every second night, for a week or two at a time, over this uneasy locality—followed, it may be, by small blisters of the size of a half-crown or so—has in our hands been generally attended with very marked benefit.

the whole case so closely resembling common continued fever, that both the stethoscopist and the non-stethoscopist are apt to be thrown off their guard."*

As a matter of course, he, who avails himself of the aid of auscultation in such a case as this, must have a great advantage over him who neglects, or is ignorant of, it; as the rational or subjective phenomena of the malady are altogether silent as to the presence of any pulmonic distress. There is one symptom, indeed—and it is often a very characteristic and most valuable one—that should always be sought for in doubtful cases; we allude to the *pungent heat of the skin* over the inflamed portion of the lung; a symptom which usually lasts as long as the inflammation continues in its first or crepitating stage.

Pneumonic consolidation of the inferior and anterior portion of the lung may exist, and yet no dulness on percussion be present, in consequence of the inflated state of the stomach or intestines. Under such circumstances, even a well-marked modification of amphoric respiration and metallic tinkling may be heard to a considerable height in the chest, and thus mislead the physician to believe that Pneumo-thorax is present.

10. In the 23rd Proposition, we find Dr. A. giving it as his opinion that "it is very doubtful whether physical examination can, in any instance, determine with certainty the evidence of simple Tubercles in the lungs." True, unless the physical examination is—as it invariably ought to be—associated with minute attention to the subjective or ordinary class of symptoms. Much, very much, may be gained from a sedulous investigation of the history of the case from the first development of the symptoms up to the period when the examination takes place, from observing the general complexion of the patient, from ascertaining the state of health of his parents and the other members of his family, whether he or any of them has been affected with hæmoptysis, &c. &c. Still we must admit that tubercular disease may exist, and to a considerable extent too, in the lungs, without giving rise to any suspicious symptoms.

11. Many will not quite agree with our author when he affirms that "physical examination cannot determine whether Pneumonia, in any of its forms, has, or has not, supervened upon Tubercles; although the prognosis in the two cases would be very different." That pneumonic attacks in phthisical cases are not unfrequently overlooked, in consequence of the comparative mildness of the symptoms, will be acknowledged by every practical physician: but it is equally true that a careful auscultatory examination may generally discover that the murmur of respiration has become feeble and mixed with a fine crepitating sound, when inflammatory action has supervened. The character of the sputa, also, is usually some-

---

* "It has been only of late years that the attention of physicians has been specially directed to the not unfrequent existence of a *latent* pulmonic affection in cases of Typhus fever. The 'subjective' symptoms of this complication are usually not very prominent or striking; and it is therefore only by the aid of the 'objective' phenomena, as disclosed by auscultation, that our diagnosis can be accurately formed."—*Schoenlein's Klinische Vorträge, in Medico-Chirurgical Rev. for October* 1845.

what altered from what it was just before the attack ; and, in some cases, the increased heat of the skin over the seat of the inflammation may serve as an additional diagnostic symptom. Let us again most strongly urge the reader never to dissociate the auscultatory from the other means of examination, nor to use the one to the exclusion or neglect of the other.

12. There is another point, in which Dr. Addison's experience has led him to a somewhat different conclusion from that generally received. According to him, acute Pneumonia not unfrequently attacks the apex of a lung, quite unconnected with, and independent of, tubercular disease.

"I have," says he, "on several occasions, known hepatization of the apex from acute pneumonia, pronounced to be phthisis by stethoscopists: they have not sufficiently appreciated the difficulty; they have neglected to inquire carefully into the history and progress of the case; and have mistaken the *pungent heat of skin* of ordinary pneumonia, for that which occurs in phthisis: and which I believe, devertheless, has often the same origin." P. 13.

And then the following case is quoted in confirmation of this statement.

"Mary B——, aged 19, was admitted, under Dr. Addison. She had always been delicate; and after the whooping-cough and measles, which she had had eight years before, had been subject to attacks of cough and cold, in which she had frequently expectorated blood.

"When admitted, she had a troublesome cough, with scanty sputa, slightly tinged with blood. There was dulness on percussion below the left clavicle, with tubular breathing and gurgling, the last extending down nearly to the margin of the ribs, where it became more dry and crepitating. Posteriorly there was dulness on the left side, extending from the apex nearly as low as the angle of the scapula; and tubular breathing and bronchophony to the same extent: slight tubular breathing and bronchophony at the right apex, with large crepitation.

"Under the impression that phthisis was present, she was ordered,

Empl. Canth. infra claviculas singulas applicetur.
Pil. Papav. c̄ Ipecac. bis die sumat.
Mist. Mucilag. ter die sumat.

Under this treatment the cough became worse; sputa more copious, fawn-coloured, and uniform, except that it contained puriform streaks; the head painful; pulse 120, compressible; cheeks flushed; skin hot and pungent; while the signs afforded by auscultation and percussion continued unaltered. She was then bled twice to eight ounces; and calomel, antimony, and opium were administered. Her mouth was kept sore by the calomel for a few days, when it was discontinued, and nothing given but julep. ammon. acet. Under these remedies the dulness, tubular breathing, bronchophony, and gurgling at the left apex, gradually diminished, and at length entirely disappeared. The tubular breathing and bronchophony at the right apex persisted longer, and caused some alarm; but these also ceased: the expectoration became white and frothy, and then, with the cough, subsided." P. 14.

We strongly suspect that tubercular disease did exist in this girl at the period of the pneumonic attack, and that eventually she would die of it. Too often, we never learn the real event of cases in hospital practice. The patients, on being relieved of their more immediate ailments, are discharged as convalescent or cured, only to sink under the more gradual advance of chronic disease.

In conclusion, we cannot but express our opinion that, whenever Pneumonia affects the upper and anterior part of the lungs alone, and is unassociated with any trace of the disease in the lower and posterior parts, there is strong reason to suspect the presence of Tubercular disease.

13. The remarks by our author on the Pleurisy that is seated low down in the angle between the ribs and the diaphragm, especially on the anterior part of the chest, are very valuable, as suggesting the necessity of great caution and the most sedulous attention that the disease may not be mistaken, as it is apt to be, for Pleurodynia, Spasm of the diaphragm or thocacic muscles, Hepatitis, &c. In the early stage of the attack, neither auscultation nor percussion can afford us satisfactory'or trustworthy information. Two or three days must elapse, before we can derive any decided assistance in our diagnosis from either. As a matter of course, however, no judicious practitioner will wait for the supervention of the physical signs of pleuritis, before determining upon his line of treatment. In all cases of doubt, where a fixed sharp pain exists, local—if not general—depletion, and the use of nauseant aperients should be had recourse to.

"When pleurisy has its seat in the parts alluded to above, it constitutes by far the most painful, and perhaps the most dangerous form, of the acute and sthenic disease. It is the Paraphrenitis of the ancients; a disease which, according to them, consisted simply of inflammation of the diaphragm. This, however, is not correct; for the pleura covering the diaphragm, is often inflamed without giving rise to the dreadful suffering observed in paraphrenitis; whereas, when acute and sthenic inflammation attacks the pleura, where it is reflected from the diaphragm to the ribs at the base of the chest; and thus involves both the diaphragmatic and costal pleuræ at the same time; then it is, that we have such intense suffering, and such an expression of agony in the countenance, as forcibly to remind us of the *risus sardonicus* of the older writers." P. 20.

14. Pleuritic Effusion, when moderate in quantity, and not associated with other morbid states of the thoracic viscera, may entirely escape detection by any means of physical examination. We cannot trust much to percussion, as the fluid does not rise high enough in the cavity of the chest, and any slight dulness of sound is, at the same time, rendered equivocal by the liver on the right, and the inflated stomach on the left, side: and even the auscultatory symptoms are only of uncertain value. A case of Bright's disease is mentioned, in which the signs of effusion into the right chest were very unsatisfactory; although, on a *post-mortem* examination shortly afterwards, it was found to contain a large quantity of serum.

15. When Pleuritic Effusion is very considerable, giving rise to unequivocal bronchophony, tubular respiration, and want of resonance and vocal vibration, physical examination has repeatedly led to a mistaken belief that these signs resulted from pneumonic or other consolidation of the lung. This mistake is more likely to be committed, when the effused fluid is partially retained in the meshes of a fibrinous exudation between the pleural surfaces. It is a fact, too, which the dispassionate stethoscopist must admit, that in some cases it is scarcely possible to distinguish the pleuritic rub or friction-sound from some of the sounds developed in the bronchi: they often resemble each other very closely; and both occasionally communicate a distinct vibration to the hand applied to the thoracic parietes.

16. After pointing out several sources of fallacy in the diagnosis of Pericarditis and of some Diseases of the Heart, we come to proposition 36, which stands thus:—

"36. A sound closely resembling a valvular murmur appears not unfrequently to be produced by the stroke of the heart against a portion of lung, interposed between it and the parietes of the chest. Under such circumstances, auscultation may lead, and I believe often has led, to the erroneous conclusion, that the heart is diseased, when it is perfectly normal in every respect.

"This sound is most frequently heard at some point in the direction of the edge of the left lung, where it overlaps the heart, to the left of the sternum, from about the second or third to about the fifth rib, and especially somewhere between the second rib and the neighbourhood of the left nipple. Its tone somewhat resembles that of a *bruit de rape;* but, at the same time, it communicates a sense of dryness and crumpling, different from the rigid squeezing or grating observed in the ordinary *bruit de rape.* It is also more variable, both in its development and its extent. We find it different at different moments, and during the different movements of the chest; and it may occasionally be made to disappear altogether, by a deep and forcible inspiration, so long as that inspiration is maintained by the individual. On the other hand, its extent or prolongation varies in different cases; or even at different times in the same case; apparently according to the extent or size of the portion of lung which happens to be struck by the heart at each systole of the organ. In a few instances I have found the sound, to a certain extent, double; the second, or that attending the diastole of the heart, being in general, perhaps, more limited and indistinct than the first. I believe this sound, which I have long observed, and now attempt to describe, to be that recently pointed out by my colleague, Dr. Barlow. It may possibly be that also noticed by Dr. Latham as frequently present in phthisis." P. 30.

17. Having cautioned his readers against deciding either hastily or very dogmatically as to the existence of cardiac disease from the presence of certain murmurs over the heart and great blood-vessels, by shewing the difficulty of distinguishing between those from a functional and those from an organic malady, Dr. Addison candidly avows, in Prop. 40, that "in certain diseases of the heart, especially when the organ is enlarged, it is difficult, or impossible, accurately to localise the murmurs, however distinct and obvious these murmurs may be." Let this unmistakeable acknowledgement from an experienced auscultator and able physician like Dr. A., serve to check the fractionally-minute and curiously-elaborate descriptions of some of the younger stethoscopists of the present day. We have again and again enunciated the spirit of the above proposition in reviewing various recent works on Heart-diseases, Auscultation, &c.

Before taking our leave of this truly practical paper, we must not omit to state that Dr. Addison has mentioned, in an early part of it, that "great contraction of the right chest after pleurisy almost as certainly draws the heart towards the same, as extensive effusion into the left chest forces it towards the opposite, side." Attention to this fact may be of great importance to the physician in forming a correct diagnosis in some thoracic maladies, when the configuration of the chest has become altered either from congenital or subsequent disease.

### II. Examples of Ptosis, with illustrative Remarks. By *John F. France.*

Mr. France is inclined to believe that, in a very great number of cases of ptosis, the cause of the paralytic weakness of the eyelid is pressure on the third pair of nerves within the skull, either from vascular fulness, or, it may be, from sanguineous or serous effusion. In not a few instances, symptoms of cerebral congestion will be found to be present; and the relief of that is often sufficient to effect the removal of the paralytic affection.

Mr. F. gives the following ingenious explanation of the much greater liability of the *motor oculi* to be so affected, than any other of the nerves of the orbit.

"This nerve, almost throughout its intra-cranial track, is in the immediate vicinity of those which must be regarded as very dangerous allies: first, hooking round the posterior cerebral artery, to traverse the narrow interval between that vessel and the superior cerebellar; then running forward nearly parallel to the posterior communicating artery, in a degree of proximity, the occasional mischief of which is demonstrated by the necroscopic examination in Case 9; and then crossing the termination of the internal carotid, immediately on its outer side, and closer to it than any other of the nerves contained within the cavernous sinus.

"The sixth nerve, it is true, is previously in actual contact with the coats of this vessel; but running along the floor of the sinus, must, in a great degree, be secured from pressure; as, from the upward direction of the current of blood, the horizontal portion of the carotid must be rather raised from, than pressed against, the inferior wall of the sinus upon each contraction of the left ventricle; and, from the same cause, the inferior wall of the artery itself must be, mechanically, the least liable to morbid distention, or rupture. That nerve, however, sometimes suffers like the third." P. 55.

That the seat of the pressure on this nerve is situated within the cranium is rendered probable from the circumstance that, being in close apposition with the 6th and the nasal branch of the 5th nerve, it is scarcely possible to conceive that one nerve alone could suffer from the supposed cause and the others escape. The double vision of the drunkard, we may remark *en passant,* is chiefly owing to a disturbance in the functions of the *motores oculorum,* produced doubtless by the distended state of the cerebral vessels; the axes of the two eyes no longer converging to the same object.

Of 13 cases related by Mr. France in illustration of his subject, No. 9 is perhaps the most interesting; the following is a summary of its chief features.

A young woman, stout and plethoric, came under treatment on May 13th for Ptosis on the right side. For a month previously, she had been suffering from headache and giddiness; and, five days before admission, she was suddenly seized with headache over the right eyebrow, accompanied by faintness and vomiting. Next morning, the right upper lid was observed to drop, and vision on the same side was found to be impaired. She was bled, leeched and purged; but without relief to the ophthalmic symptoms. The pupil of the affected eye was much dilated, and imperfectly mobile; the movements of adduction, elevation, and depression of the ball were more limited than natural, but that of abduction was complete.

She was ordered a grain of quinine three times a day, and five grains of Plummer's pill night and morning.

On the 15th, she was not so well; the Ptosis had become complete; and the three motions of the globe, and that of the pupil, were more restricted. The quinine was suspended, and one grain of calomel with a quarter of a grain of opium* ordered to be taken three times a day: a blister also was applied to the nape of the neck. In the course of three days, the gums began to be sore; but no decided improvement in the movements or sight of the eye; the right pupil remained much dilated. On the morning of the 31st, she was suddenly seized with apoplexy, and died in the course of a few hours.

On *dissection*, there was discovered a recent extravasation of blood at the base of the brain; it had come from the posterior communicating artery of the right side. Resting on the aperture in this vessel, was a circumscribed solid coagulum, and under this was the right motor nerve spread out, having obviously undergone considerable compression.

In cases of prolonged Ptosis, the contractility of the pupil of the affected side is apt to become more or less seriously impaired. This is just what we might expect, considering the connexion of the third nerve with the Ciliary Ganglion. The dilatation of the pupil so induced is apt to make the oculist suspect the presence of Amaurosis; and this mistake is the more liable to be committed, seeing that considerable dilatation of the pupil, from whatever cause it may arise, is almost always attended with greater or less indistinctness of vision. The concluding remarks of Mr. France will be read with interest, alike by the physiologist and practical physician.

"Loss of power of the motor-oculi nerve ensures, as certainly, a direct loss of the healthy contractility of the pupil, as it does of that of three recti, of the levator palpebræ, and of the inferior oblique muscles.

"This is a fact, indeed, which has been abundantly demonstrated by the experiments of physiologists; but it has not, I believe, received the confirmation of a detailed series of cases before. That now presented, while giving pathological testimony to the controlling power of the third nerve over the pupil, shews, at the same time, that this power is exercised only, as anatomy would indicate, by the inferior division of the nerve. With respect to the mechanism of the pupillary actions—amid the conflicting results of microscopical examinations, which, on this subject, just cancel one another—we are still fain to resort to rational argu-

---

* We may take this opportunity of expressing our objection to this too-frequently used combination of Calomel and Opium in almost all cases indiscriminately, when the design is to mercurialize the system. In cerebral affections, and in other diseases where there is a tendency to cephalic disturbance, the action of the opium is apt to be most injurious. The Hydrargyrum cum Cretâ combined with carbonate of Soda or with the pulv. Cretæ Comp. (according to circumstances)—in doses of from 3 to 5 grains of each repeated every four or six hours—will be found a most safe and useful substitute.—*Rev.*

ments; and, among them, this obedience of the pupils to the third pair does appear to me to constitute one of the most forcible in favour of the muscular theory." P. 63.

III. Reports of the Clinical Society, from January, 1845, to March, 1846. By *Alfred Poland:* Surgical Division.

Our extracts from this article will be very short. The cases are not reported with due care, nor are their relations to practice sufficiently dwelt upon, to entitle the report to much notice. How comes it to pass that none of the surgeons of the hospital revise it before publication, and incorporate with its details such reflections as their perusal must enable *them* to suggest? This matter requires attention, if the Guy's School wish to maintain the well-merited reputation of their " Hospital Reports."

A. There were two cases of Traumatic Tetanus; both proved fatal. In the first, the symptoms were of a well-marked hydrophobic character; for the patient " had trismus, opisthotonos, difficult deglutition, abhorrence of fluids, great pain, spasms every quarter of an hour, &c.; he was quite sensible." Death occurred three days after the invasion of the disease, which had been induced by a severe injury of the fore-finger. In this case, as well as in the other which occurred in a young child, the nerves at the seat of the injury were found to be *inflamed.*

B. Under the head of Injuries and Diseases of the Lungs and Appendages, is briefly related a case of a Foreign Body, a sixpence, introduced into the Trachea. " No symptom was observed, but an occasional spasmodic cough. Conjectures were raised as to its position, and even as to its presence. The stethoscope elicited a metallic sound. Tracheotomy was urged by some, but considered unadvisable; and in the night, during a paroxysm of coughing, the man ejected the coin, and was soon presented in good health."

C. The external Iliac artery was tied in a case of Femoral Aneurism. A fortnight after the operation, secondary hæmorrhage supervened; and, as this recurred on several occasions, the patient gradually became exhausted, and died on the 32nd day after the ligature had been applied.

On *dissection,* " the Iliac artery was found divided ; its *upper* end plugged with coagulum ; its *lower* end quite open, and devoid of clot."

D. The following is surely a *cas rare:*

" Acute Phlebitis occurred in a young man, aged twenty-five, of bad health and constitution, who had received a kick on the arm, to which leeches had been applied; phlebitis ensued, with severe constitutional symptoms; a livid appearance of the skin in the neighbourhood of the blow ensued; an incision was made, *and a dead leech found embedded!* No wound, except a leech-bite, could be observed externally to account for its entrance. The man, however, died exhausted; and no autopsy was allowed." P. 69.

In a case of Chronic Phlebitis, which supervened upon opening an abscess near the right ancle, and in which an œdematous state of this extremity, and also of the scrotum and prepuce supervened, " the vena cava inferior, and right iliac vein were found obstructed by coagula adherent to the inner coat; the left iliac vein was contracted; the kidneys were granular, and the lungs were affected with old pneumonia."*

E. A case of Rupture of the Bladder occurred in a man, from another butting his head in the patient's left groin, and throwing him a summersault over his back. The urine was drawn off at first by a catheter; subsequently it was passed with freedom and in a healthy state. Symptoms of peritonitis came on, and the patient died in 90 hours. On *dissection*, "the bladder was found ruptured to the extent of three-quarters of an inch at its base, and some urine was extravasated behind the peritoneum to near the lumbar region."

F. There were 26 cases of Hernia; 13 male and 13 female. The latter were all femoral,† as also 3 of the former; the remaining 6 were oblique inguinal. In several, the reduction was effected by the taxis, aided with the warm bath, and the application of ice to the tumour. The mortality after the operation for Hernia in hospitals is almost always considerable, in consequence of its being injudiciously delayed and, probably also, of unnecessary force having been used in the taxis, previous to the admission of the patient.

G. A case of Obstruction of the Large Intestine, of five days' standing, is related; the symptoms do not appear to have been alarming. The patient was affected with a reducible scrotal hernia and also with *scirrhus* of the rectum. "A flexible bougie was passed per anum to some extent, as also a long flexible tube; enemata were given, and calomel and opium administered, but to no effect: the man died suddenly on the third day collapsed. The autopsy shewed extravasated fæces, and an open ulcer of the rectum about the size of a shilling."

The practice in this case seems to have been anything but wise and proper; indeed, we strongly suspect that the mechanical interference accelerated its speedy close. When will medical men learn to do nothing, when there is no rational prospect of doing good?

H. In the following extraordinary case of Ovarian Disease, nature seems to have effected a cure. The tumour was of three years' standing, when the patient was admitted into the hospital, and it had been tapped about six months before, and three quarts of fluid drawn off.

"It then gradually increased, and attained the size of thirty-seven inches, measured round the body at the umbilicus. She complained of a good deal of pain: the catamenia continued regular. On admission, diuretics were first had

---

* In the Medical Clinical Report is related a case of fatal Pleuritic Effusion (the pericardium also was full of a dark fluid), in which on dissection the *left* vena innominata was found totally obliterated, being converted into a dense cord; "the *right* one had slight recent obstruction; in the right jugular vein there was a coagulum, solid but recent; vena azygos free. In the superior cava was a pedunculated growth, about two inches in length."

The occurrence of such cases as this suggests the utter inutility, or rather impropriety, of active treatment, when much obscurity hangs over the diagnosis.

† As stated in this Journal for last April, M. Malgaigne denies that femoral hernia is more frequent than inguinal in women. We are not, however, aware that any other surgeon has expressed his concurrence with M. M.'s opinion on this point.

recourse to, and with benefit; but at the end of six months, the tumour beginning to increase, she was tapped, and two ounces of grumous blood, like the contents of a hæmatocele were drawn off. Symptoms of peritonitis supervened. An abscess formed in the abdominal parietes, which was opened, and afterwards assumed an unhealthy action; large pieces of slough came through the opening; and on one occasion a piece about two feet long was removed, and appeared to be nearly the whole of the ovarian cyst. The woman became exceedingly low; but, under tonics and stimulants, recovered, and left the hospital, about a year afterwards, perfectly cured. In the course of the following twelvemonth, while in the country, she was seized with bronchitis and symptoms of phthisis, and died. Unfortunately an inspection was refused." P. 81.

Perhaps it may be as well to allude here to a case of Tumour (subsequently related) on the front of the neck, in the situation of the Thyroid gland, of which it was supposed to be a malignant growth. "It increased rapidly, and impeded respiration; fluctuating at one part, it was tapped, and much fluid drawn off with relief. The tumour became inflamed and sloughed, large masses coming away, until the whole was removed; the patient required much support."

I. Passing over the cases of recent Fractures, it may be worth while to allude to two cases of Ununited Fractures which occurred. One was of the humerus, of eight months' standing: *there was an excess of the phosphates in this patient's urine.* As a variety of methods had been tried, but without avail, amputation of the extremity was recommended; but the man would not consent.

The other was of the tibia and fibula, of nine months' duration, and was owing to the imperfect union of a compound fracture. The starch bandage was applied, and Iodine given internally; the union became complete.

J. We much fear that the following record of 9 cases of Scirrhus of the Mamma is, from the off-hand air of confidence with which a cure is spoken of, apt to mislead the young reader as to the *real* results of operations in this disease.

"Of these cases, four were cured by the knife, in one of whom the operation nearly proved fatal, owing to a very severe attack of erysipelas; and in one of these cases, also, the disease had formed a large, open, fungating sore, discharging a profuse fætid serosity; but the glands, with the axilla, were free, and thus so far the operation warranted. Four were unrelieved; two from an unwillingness to submit to the operation, and two from the advanced state of the disease, the neighbouring glands being extensively involved. The last was a case of recurrence of the disease after an operation which had been performed eighteen months previously; the patient was in bad health and the disease in an active state, rapidly increasing, and producing intense pain and suffering: she lingered on for three months, and then sank from exhaustion." P. 132.

The last case is but a sad comment on the cures (!) said to be effected by the knife in the four first.

K. Among the cases of Dislocation we find that two proved fatal.

"One was a very curious and rare dislocation of the astragalus, with the tibia and fibula (the ankle-joint being entire) off the os calcis and os naviculare, occasioned by a fall of thirty feet down the stairs of a dock basin. The precise condition of the position of the bones was not clear: reduction was attempted, but to no avail: sloughing of the integument supervened, and the articulating surface of the astragalus exposed. The patient's constitution began to suffer, and amputation was had recourse to on the seventh day : the stump sloughed, and fatal prostration ensued. The tendons about the ankle were found much displaced: the tibialis posticus was thrown in front of the tibia; and the flexor longus pollicis in the interosseous grove on the under surface of the astragalus : the vessels were entire : the posterior tibial nerve was much stretched over a sharp edge of bone. The other dislocation was also of exceedingly rare occurrence, and its nature was only ascertained after death : it was that of the sacrum thrown forwards from off the ossa innominata. It was produced by a sudden blow, on the lower part of the spine, by the buffer of a railway-engine. The man was brought in insensible, with a severe scalp-wound, and fractured ribs and emphysema. He lived several hours. The *sectio-cadaveris* displayed, besides the dislocation above alluded to, a fracture of the spine in the dorsal region." P. 134.

We fear that injudicious force was applied in attempting to right the foot in the first of these cases. There is often vast mischief caused in this way.

The Medical Clinical Report is drawn up with even less care than the Surgical. For example, under the head of Diseases of the Organs of Circulation, we read that there were 4 cases of Aneurism admitted, and that, of these, two were relieved and two proved fatal. "Of those reported relieved, it becomes a question whether they were really aneurism. The age of one was twenty-six : there was a rough murmur heard over the aorta; and much pain between the shoulders. The age of the other patient was forty-two ; there was no murmur present, but the heart's sounds were prolonged. Both these patients had been much exposed to cold, and both had been the subjects of rheumatism." What ?—are we to be told nothing more of the symptoms of a case of alleged aneurism than that "the heart's sounds were prolonged?" Again, we are not informed whether a correct diagnosis was formed in any one of the four cases; from the imperfect statement of their histories, we suspect *not.*

B. In addition to the cases of chronic Phlebitis and obstruction of veins, already noticed, we may here mention that there was admitted, under the care of the physicians, "an interesting case of obstruction to the venous circulation of the upper half of the body. The head and neck appeared bloated and ready to burst ; the lower half of the body was of the normal size and appearance. It was conjectured that some tumour was pressing upon the superior vena cava. The man left with but slight alteration."

In a case of ascites which proved fatal, "the inferior vena cava was found filled with a fungoid mass, even to the entrance of the auricle; the vena azygos was much enlarged."

It is obvious, therefore, that obstruction of the internal veins is by no means of infrequent occurrence.

C. A case of Cancrum Oris—successfully treated by the application of pure nitric acid to the sloughing ulcer, and the internal exhibition of the *chloras potassæ* with *decoct. cinchonæ*, wine, eggs, beef-tea, &c.—is recorded.

D. An example of Scirrhus of the Pylorus, in which the symptoms were chiefly palpitation of the heart and dyspnœa, while those connected with the stomach were so indistinct as seem to have scarcely attracted any notice, is narrated. The patient died of an attack of diarrhœa. On dissection, "the stomach was distended with fluid, and at its pyloric extremity a scirrhous enlargement existed of about the size of an egg: the passage from the stomach to the duodenum was consequently constricted, but not to such an extent as to prevent the transmission of its contents."

### IV. Clinical Report of Cases admitted into the Petersham Ward. By *John C. W. Lever*, M.D.

This report contains the account of 74 cases treated by Dr. L. in the female or obstetric ward during twelve months, from June 1845 to June 1846. The cases are much more carefully drawn up, and the information is greatly more satisfactory than in the preceding reports of the Clinical Society.

A. Under the head of Ovarian Dropsy, is related a case of Fungoid Disease of the Uterus and left Ovary, and involving more or less all the pelvic viscera. During life, fluctuation had been felt distinctly per vaginam, as well as per rectum; and accordingly with a trocar, introduced into the former passage, a puncture was made, and a dark-coloured fluid was evacuated. She continued better for about four months afterwards, when it was again deemed necessary to repeat the operation, and about 12 oz. of a lightish yellow-brown fluid were drawn off. She was seized with diarrhœa a week or two subsequently, and died. On *dissection*, the disease of the uterus was of two kinds, "the soft medullary fungus, and the hard cartilaginous scirrhus; and a point of special interest was the development of cysts, containing clear serous fluid, and having the cerebriform fungoid masses projecting from the walls: such an one was found at the post-mortem examination, and was doubtless similar to that which had been punctured during life, and the walls of which had subsequently taken on a sloughing action."

Dr. Lever frankly confesses that he was mistaken in his diagnosis of this csse; having regarded it, during the life of the patient, as one of encysted Dropsy of the ovary. He omits to tell us, by the bye, whether the fluid, discharged by the operation, was examined with the microscope; but the following is the account of the microscopic appearances observed on examining the parts after death. "The soft medullary tumours were made up of cells, having thick walls, giving them a double outline, and containing one, two, or three nucleated cells: there seemed to be no connecting tissue, which accounts for its soft and cerebriform character. They may be taken as a type of malignant formation."

With respect to the treatment adopted, Dr. L. remarks:—

"I conceive that it was the best that could have been employed under the circumstances; and, in my opinion, interference was necessary; for the fæces, when solid, were of tape-like form; and, as the patient had but slight control over the sphincter-ani, they were, when fluid, frequently passed before she could reach the water-closet. The bladder and urethra were subjected to undue pressure and irritation; a catheter could with difficulty be introduced; the nerves were injuriously acted upon; there were numbness and weakness of the right lower extremity, causing her to limp. On each occasion when the fluid was drawn off, she recovered the perfect command of the sphincter ani; the calls to void the urine were less frequent; the numbness and weakness of the right leg were lost; and she had as perfect control over its movements as over those of the left." P. 194.

B. Two interesting cases of Pelvic Abscess—both in the *left* iliac fossa —are recorded. In one, the abscess burst into the gut and was discharged per anum; in the other, it was opened externally, and a quantity of pus, mixed with fætid gas, was evacuated. Both patients did well.

C. Two examples of Vascular Tumour of the Urethra in the female are related. As this malady is apt to be overlooked by medical men, and is perhaps scarcely known to many of them, it will be useful to mention briefly the particulars of one of the cases.

"A woman, æt. 67, had been obliged to relinquish her occupation as a sempstress in consequence of severe pain in the urethra, irritability of bladder, and constant desire to void her urine, which she attempted every five minutes; difficulty, but no pain, was experienced in passing her motions. After the trial of various popular remedies, as well as submitting herself to the care of surgeons, she applied at a dispensary at the west-end of London: there the cause of her sufferings was detected. Her general health was tolerably good, and there was no discharge of any kind at the time of her admission. She had constant calls to void her urine, the passage of which occasioned her great pain, occasionally of a shooting character, extending upwards to the abdomen, outwards to the hips, and backwards to the perinæum. On separating the external labia, a vascular tumour was seen protruding through the meatus urinarius, of a florid red colour, and about the size of a kidney bean. On minute examination, which was with difficulty permitted, on account of the exquisite sensitiveness of the growth, it was found that it passed some little distance into the urethra, and was so vascular that it bled upon the slightest touch: the suffering occasioned by the examination was considerable.

"The patient was placed on her back, and the tumour, with the lining membrane of the urethra attached to it, was removed. The nitrate of silver was then applied, and she was desired to keep the part constantly wetted with white lotion." P. 219.

The operation caused much pain, which continued all night. The nitrate required to be applied, at intervals of three or four days, several times. At length, in the course of about a month, a cure was effected.

D. We regret to observe that, in one case of Scirrhus of the Os Uteri, Dr. Lever thought it "advisable to make the experiment of removing the diseased portion. This was accomplished by Mr. Hilton, who, failing to draw down the uterus so as to protrude the os uteri through the external parts, removed the disease with this organ *in situ*. Considerable hæmorrhage attended and followed the operation, but was arrested by a large and firm vaginal plug. The operation was followed by peritonitis, which was treated by repeated leechings and the administration of calomel, antimony and opium. When convalescing from this, she was attacked with dysentery of a most aggravated and fearful character, and for some days her life was in extreme peril; but she slowly recovered, and left the hospital to undertake the duties of a turnkey at one of the metropolitan prisons. The womb, however, was not freed from disease; for on making an examination some time after she had been performing her public duties, I found the discharge copious and offensive, with a return of the scirrhus."

E. A number of cases of Simple Ulceration of the Cervix Uteri are related. All were relieved or cured by the application of the nitrate of silver, &c. through a glass speculum, every second or third day, and attention to the general health. Even in instances where the ulceration was of a more unhealthy, and even a very suspicious, character, much benefit was obtained from the occasional application of pure nitric acid to the seat of the disease, and the internal administration of alterative tonic medicines, along with nourishing diet, &c.

V. On the Physiology of Cells, with the view to elucidate the Laws regulating the Structure and Function of Glands. By *Thomas Williams*, M.D.

The great object of the author, in this elaborate paper, is to endeavour to determine the true laws regulating the organisation and function of the Liver, by tracing its development and condition through the various classes of animals, from the simple *Actinia* up to the highest order of the *Vertebrata*. The comparative anatomist will not fail to appreciate the value of the minute details and numerous illustrative wood-cuts which Dr. Williams has given; but they are quite unsuited to the pages of this Journal. Indeed, some readers may be tempted to ask, how it comes to pass that a paper like this should find a place in the Reports of a Hospital? We shall give but one extract, as it bears upon some disputed points in General Physiology.

"From the description of all preceding observers of the structure and peculiarities of these humble organisms, it was confidently expected that the biliary system in the actinia would be discovered under circumstances of the greatest practicable simplicity,—under the form, namely, of 'a follicle' in the walls of the digestive sac. Practical inquiries will indisputably prove that such statements are founded only on theoretical suppositions. Each succeeding author on comparative physiology, has at once admitted and transmitted the errors of his predecessor; so that it has actually become an anxiom, that, however inextricably complex an organ may be in the highest orders of animals, its remote submultiple, its rudest dawning in the distant extremity of the declining scale, will be found, without a solitary exception to diversify the monotonous law, to consist of a follicle. This is the invariable terminus of all researches after the 'analogous' and morphological reductions of organs, which acquire complexity of internal arrangement. In regard to the provisions made for the supply of secretions in animals, of which the body altogether is no more involved than a large follicle, it seems superfluous to urge the argument, that, if a follicle, with its component machinery of cells, membranes, and vessels, were furnished for the exclusive purpose of elaborating a single secretion, a subordinate organ, subservient to uses of a minor importance, would exceed, in complexity of structure, the organism viewed as a whole. In the minute structure of the actinian polype direct proofs may be obtained to confirm the view, that a follicle is not the ultimate link in the chain of morphological reductions. Aggregations of nucleated cells occur in the depressions between the vertical duplications of the digestive membrane, which in their intimate organization, seem, in a very obvious manner, to unite all the requisite of a gland. These cells are held together by, and are lodged in, a semi-fluid plasma of extremely tenacious property;—a property which acquires importance in the use apparently assigned as one of its functions of holding the the cells *in situ*, and of preventing displacement and injury during the dilatations and contractions which the stomach is destined to undergo." P. 282.

VI. The case of "*Supposed Spontaneous Perforation of the Stomach terminating successfully,*" related with great minuteness by Dr. Hughes, is one which will be viewed very differently by different readers.* That the symptoms, which usually attend sudden rupture of some part of the alimentary canal, were present in the present case cannot be disputed; and the treatment adopted by our author, on the suspicion that this most alarming lesion existed, was the very best that could be followed.

"Opium, quiet, and starvation were the three remedies employed. The opium was administered in moderate doses only, as they appeared to have the desired effect; and those doses were diminished as soon as the symptoms appeared to justify the reduction. In the first twenty-four hours the patient took between seven and eight grains of the drug, without any of the ordinary effects of opium upon the brain, the iris, or the tongue, being at any time noticed. This fact itself appears to indicate that some very severe disturbance existed in the system. After the first twenty-four hours, the patient took only four grains in the day and night, and this quantity was soon reduced to three grains. By the mouth she took no other medicine of any kind. Warned by the unfortunate experience of Dr. Stokes in one of his cases, it was determined to administer no aperient. On the seventh day the bowels acted spontaneously.

"For a period of eighteen days the patient was not allowed to move or to be moved, in the slightest degree, from the supine recumbent position. When the bed became hard and uncomfortable she was removed in a sheet, without any alteration of her position, to another already prepared for her. The enemata were administered as she lay upon her back.

"For a period of forty-eight hours the only sustenance allowed her was two teaspoonfuls of toast-water, given every hour." P. 341.

Nourishment was given by the administration of beef-tea enemata.

Dr. Hughes very judiciously cautions the reader against the use of stimulants and purgatives in all cases, where there is the slightest ground for apprehension that perforation of part of the alimentary canal either is imminent, or has taken place. We are inclined to extend the same advice to very many cases of Ileus, or Obstruction of the Bowels, when there is any tendency to inflammatory symptoms supervening. A vast deal of mischief is continually done by persisting in the use of powerful doses of drastic purgatives, when the bowels refuse to act after 24 or 36 hours' administration of those in ordinary use. It is a dangerous error to attribute the constipation to mere sluggishness or torpor of the intestinal tube. There may be, if not mechanical obstruction, spasm or incipient inflammation; and certainly violent drastics are not the best or safest remedies for the treatment of either of these morbid states.

VII. The case of "*Ulcer of the Stomach, leading to Perforation and Death in 19 hours,*" related by Mr. Ray and Mr. Hilton, exhibits several points of resemblance to the case of supposed rupture just given. Both patients were young women, the one 28 and the other 27 years of age;

---

* Does not the circumstance of the catamenia having appeared within 60 hours from the invasion of the alarming symptoms, and continuing to flow for two days as in health, argue somewhat against the idea that so serious an accident as rupture of the stomach was present?

and both had been suffering from symptoms of stomach ailment. In both, the attack of pain in the epigastrium was sudden and so severe as to occasion alarming prostration. (We had written so far before we discovered that the patient was the same in both cases; the second and fatal attack occurred four months after the first; she survived only eighteen hours.)

*Dissection.*—On opening the abdomen, a quantity of turbid fluid, containing portions of gooseberries, cherries and strawberries, in addition to very numerous small shreds of soft lymph, was found extravasated among the viscera. Besides the appearances of very recent inflammation, there were extensive old cellular adhesions of the stomach to the liver, and of the stomach and intestines to the abdominal parietes.

"The stomach presented a central constriction, known as the hour-glass contraction of the stomach, but not in an extreme degree. A little gentle pressure upon the stomach, caused some air and fluid to escape from amongst the old adhesions at the smaller curvature of the stomach, and so discovered to us the abnormal opening from it into the peritoneum, about midway between the œsophageal and pyloric apertures. A long incision was then made through the anterior wall of the stomach near its lower edge, parallel with the larger curvature; and on turning this part upwards the contents of the stomach were seen, composed of cherries, strawberries, and gooseberries, scarcely broken. These were aggregated at the pyloric extremity of the stomach, and completely filled it. The abnormal opening from the stomach at its smaller curvature was also immediately seen, occupied by a piece of a strawberry."

"The mucous surface of the stomach was healthy, except at about midway between the œsophagus and pylorus (at which parts the circumference of the stomach was diminished, corresponding to the external hour-glass-like constriction), and towards the smallest curvature, where it presented the rugæ of the mucous membrane radiating towards the pylorus from a small surface, which had all the appearance of a cicatrix of an old ulcer: half an inch below this part was seen a recent ulcer, about the size of a fourpenny-piece, irregular in outline, its edges unequal in thickness, and highly vascular: it had extended through the mucous membrane, the sub-mucous fibrous structure, as far as the muscular fibre. On looking to the peritoneal surface of the stomach corresponding to the internal position of this ulcer there was no evidence of any recent inflammation. About half an inch above the cicatrix, to which reference has been made, was placed the base of the conical canal, passing upwards and backwards obliquely through the stomach, which had allowed the escape of some of its contents into the peritoneum, and so caused the death of the patient." P. 346.

Mr. Hilton is of opinion, from the consideration of all the phenomena exhibited on dissection, that "the perforating ulcer, which caused the patient's death, was the same which induced her previous illness; and that the peritoneal opening became closed by the deposition of adhesive matter around it, fixing it to the nearest organ, the liver; and that the occlusion was complete up to the period of the beginning of the recent fatal symptoms, when some of the old adhesions were detached or broken through, from the distension of the stomach by a large quantity of fruit, or possibly by some other mechanical cause, which does not appear, and so allowed the second escape of the contents of the stomach into the peritoneum. This latter is the explanation at which I arrive, because the cicatrix, to which reference has been made, does not extend through the entire thickness of the walls of the stomach, which negatives altogether the idea of perforation at that part: and, further, from the fact that there was seen, at the inspection of the body, a thick layer of old lymph, in part detached from the peritoneum, over the opening, allowing of an escape through it, but which, during its super-position, as regarded the opening, would have as completely prevented any occurrence of the kind."

VIII. The article on certain "*Appearances in the Stomach after Death,*" by Mr. Wilkinson King, is written in such a strange pedantic style as to be scarcely intelligible. Our readers would not thank us, we are sure, for any extracts: and we therefore at once proceed to notice the "*Digest of 100 cases of Chorea, treated in the Hospital,*" by Dr. Hughes.

Of these 100 cases, 73, or nearly three-fourths, occurred in females. The greater nervousness of constitution in girls and their consequent greater susceptibility to all external impressions, and especially to one principal exciting cause of the complaint, Fright, will partly account for this very marked preponderance. The changes, too, induced in the female system on the approach, and from irregularities, of the catamenial secretion, may have something to do with it.

In three of the cases, the sudden suppression of the menses, resulting from fright, is mentioned as the immediately-exciting cause of the attack. "I believe," says Dr. H. "it will be found that females who have passed the age of puberty are rarely affected with chorea, unless they be troubled with some irregularity of the periodical function of the uterus, or unless the complaint be clearly connected with rheumatism, or with disease of the brain or spinal-marrow."

*Age.*—Of the 100 cases, 33—11 in males and 22 females—occurred at or below the age of ten years; 45—11 in males and 34 in females—between ten and fifteen; and 22—5 in males and 17 in females—above the latter age. Above eighteen years of age, the number of males affected was the same as that of females, viz. 4. It may be also worthy of notice that the two youngest patients, aged five and six, were males.

As to the *alleged exciting causes*, it is difficult to speak with any degree of confidence. 31 cases are referred to fright; and 8 to rheumatism, simple or complicated. We extract the remarks of our author on the connection of Chorea with Rheumatic Affections.

"The connection between the two diseases has been often noticed; and the frequent occurrence of spasmodic affections with pericarditis, which, in the great majority of cases, is of a rheumatic origin, has been particularly illustrated by Dr. Bright, and, more recently, by Dr. George Burrows. It appears at least doubtful whether, in most of such cases, there exists anything more than a sympathetic affection of the spinal marrow; seeing that after the removal of the rheumatic affection the chorea is usually curable by the same remedies which are found available in cases of chorea having a different origin. There are, however, some exceptions, in which the membranes of the cord seem to be inflamed and thickened, and in which local depletion, counter-irritation, and the continued action of mercury, appear to be the means more especially calculated to remove the complaint, which, in such instances, is often very rebellious.

"Among the fifty-eight cases in the table in which, after inquiry, either no exciting cause could be ascertained, or a particular cause of the complaint is mentioned, eight are enumerated as having their origin more or less directly in rheumatism. This number, amounting to nearly 14 per cent., may be, perhaps, regarded as a fair average." P. 376.

In some of the cases of rheumatic complication, there was Pericarditis present.*

*Treatment, mode and duration of.*—With respect to the latter point, it would seem that, in an immense majority of the cases, the patients were under treatment for from three weeks to two months. We shall now briefly notice the results of the principal modes of practice that were tried.

To trust entirely, or even mainly, to the use of *Purgatives*, in conjunction with good diet, and, it may be, wine also, as some have advised, is a practice that cannot be favourably spoken of. There is a form of tonic aperient however, which may often be used with very great advantage, more especially in the weak, ill-fed children of the poor. This is a cold infusion of Rhubarb (ʒss. sliced) in port wine (℥viij); two or three table-spoonfuls may be given thrice a day.

"The effect of this medicine, together with good diet, is often very remarkable; not only upon the disease for which it is prescribed, but also upon the health, strength, and general appearance, of the little patients. It must be, however, acknowledged that, while it improves the general health, it sometimes fails in curing the complaint." P. 383.

We need scarcely say that the state of the bowels must be carefully attended to in every case of Chorea, without exception; as the irritation of unhealthy secretions, worms, &c. is well known to aggravate every spasmodic disease. But this is a very different thing from following the purgative plan of treatment.

*Mineral Tonics.*—Different practitioners have given a marked preference to different sorts, and different preparations, of this class of remedies.

"While one regards Arsenic as a specific for true chorea, another cures nearly all his cases with the sulphate or the oxide of Zinc; and another considers the sulphate or carbonate of Iron as an almost infallible remedy. This may perhaps arise, in a great measure, from habit. It is possible that all may be nearly equally efficacious. I have seen each of these, and many other remedies, occasionally cure the complaint; and I have seen them all occasionally fail. I have seen zinc cure after arsenic and iron had failed; and iron cure after zinc had failed. Iron sometimes acts with excellent effect, and with great rapidity, after zinc has been administered for weeks, and in large doses, without any, or scarcely any, impression being made upon the disease. It also frequently acts exceedingly beneficially in improving the general health, and completing the cure, after that has been, in a great measure, effected by the administration of zinc." P. 385.

According to Dr. Hughes' experience, *Arsenic* is not so useful as other

---

* Whenever there is any tendency to Chorea or other Spasmodic affections, the debilitation of the system has invariably a tendency to aggravate the complaint. There cannot, we think, be a doubt but that the condition of the circulating fluids has a good deal to do, if not with the induction, at least with the persistance, of Chorea. As far as we know, medical writers have not yet ascertained with sufficient precision whether cardiac or vascular murmurs are generally present or not, independently of any rheumatic complication; and whether, if present, they subside as the patient gets well.—*Rev.*

mineral medicines; but he has not used it in many cases. *Iron* is especially well suited to chlorotic females, to girls approaching the period of puberty, and to anæmic children. It was usually given in the form of the sulphate with extract of gentian, the sesqui-oxide, and the compound steel mixture. *Zinc* was more largely administered than any other metallic preparation, and usually in the form of the sulphate. "Beginning with one or two grains three times a day, it has usually been gradually increased by a grain to each dose at each visit twice or thrice a week; or by adding one grain daily to the whole quantity taken in the day. It has been given either in the form of pill or in solution. The dose has been increased up to thirty-six grains three times a day. It has not often caused sickness. The stomachs of some persons, however, appear unable to bear it even in small doses; and in others the organ rebels against its increase after a certain number of grains has been attained."

| | | |
|---|---|---|
| " Zinc was prescribed in .. . .. .. | 63 cases | |
| Of these, it effected a cure in.. .. .. | 45 .. | or 71 per cent. |
| it relieved in .. .. .. .. | 2 .. | |
| it failed to effect a cure in .. | 16 .. | or 25 per cent. |

" In seven of the forty-five cases iron had previously failed; the zinc was given together with iron in one, and together with the administration of electricity in five cases. In two cases the zinc was given in the form of oxide." P. 387.

*Electricity.*—The effects of this remedy in Chorea are sometimes remarkable. Occasionally it will effect a cure, and this too rapidly, after a great variety of remedies has been tried in vain; in other instances, it does harm rather than good. " The cases in which it appears to be more especially applicable are those occurring in young women, in whom the disease assumes somewhat of an hysterical character, and those protracted cases in boys in whom other remedies have been tried ineffectually, and in whom the disease is dependent upon no obvious source of irritation, and has an injurious effect upon the general health instead of being affected by it."

*Shower Bath.*—Dr. Hughes rather oddly remarks that he is not able to judge of the influence of this remedy, when employed *alone* in the treatment of Chorea. Who would ever be so mad as to do this?—except indeed some of your French *experimentateurs*, determined at all hazards to dress up some statistical table or another, in the way of a report!

Shower bathing is certainly not suited to all cases. Much will depend upon the circumstance whether the young patient likes it or not, and also whether a feeling of general warmth is induced after its use. It is therefore to be regarded as a mere auxiliary, but never as the principal remedy.

*Results.*—Of the 100 cases, the general result was—

| | |
|---|---|
| " Cure in .. .. .. .. | 80 cases |
| Almost perfect cure in .. .. | 7 " |
| Relief in .. .. .. .. | 6 " |
| Little improvement in .. .. | 4 " |
| Death in .. .. .. .. | 3 " |
| Total . .. | 100 " |

Of the 3 fatal cases, one occurred in a young woman, 17 years of age, and was accompanied with suppression of the menses; in another, death was the result of an attack of Pericarditis, in a girl 16 years of age; and in the third, which occurred in a man, 25 years old, the malady appears to have been connected with disease (although not extensive or well-marked) in the cerebro-spinal axis.

The last patient occurred in Dr. Hughes' practice at the hospital, about two years ago. Without any obvious cause, the patient became affected with twitchings in both hands; in the course of a few days, these irregular movements extended to the arms; next to the face, and organs of articulation; then to the legs and to the trunk; so that, when admitted, he was in a lamentable state of jactitation in every part of the body. The mouth, eyes, trunk and limbs were twisted and turned in every possible direction, without a moment's cessation. The motions and contortions were so violent as to throw him out of bed, even though boards were put at the side for his protection. He was all this while perfectly sensible; nor was there paralysis of any part. Tonics and generous diet were ordered. He did not sleep a wink during the night, being in a state of constant and violent motion the whole time. Next day, he was much exhausted; and, as the convulsive movements continued as severe and incessant as ever, it was manifest that, unless sleep could be procured, he could not last long. Opium was given repeatedly, but with no effect. He died about 40 hours after admission into the hospital.

The report of the *dissection* is as follows:—

"Head: a small quantity of blood was spread thinly over the arachnoid, on both sides of the vertex; but it was doubtful if this might not be the result of violence in removing the calvarium. The edge of the fornix on the right side was much softened; and the surface of the third ventricle tumid, red and soft. No other morbid appearance was observed in the brain. Spine: the rachidean fluid was opaque, yellow, and largely coagulable by heat, and the medulla, upon section, was thought to be softer than natural. Chest: evidence existed of slight pleuritis and pneumonia. The blood was thick, dark, and fluid. Patches of ecchymosis existed below the attached pericardium and the endocardium. The lining of the aorta was dyed by the fluid blood, and its root sprinkled with atheroma." P. 392.

Whether our readers will agree with Dr. Hughes in regarding this anatomical inspection as "especially valuable," we leave to themselves to decide. The case is a remarkable one of fatal Choreic Convulsions. *A priori*, we should certainly expect the Spinal Marrow to have been the seat of the chief pathological changes: but, alas! morbid anatomy throws but little, if any, light upon too many of the Neuroses.

### IX. Cases and Observations in Medical Jurisprudence. By *Alfred Taylor*, F.R.S.

As Mr. Taylor has so deservedly acquired a high reputation for skill in medico-legal enquiries, more especially in those which relate to Death from Poisoning, and as, most unfortunately, instances of this description seem to be on the increase each successive year, it is highly necessary for medical men to be fully prepared for the performance of those duties which moral, as well as legal, justice requires of them. This can only be done by having their attention frequently drawn to the narratives of the more important cases as they occur. We shall therefore briefly notice those which Mr. Taylor has recorded in this paper.

1. The first case is one of fatal Poisoning from Sulphuric Acid. The sufferer was an infant, only four months old. The medical man first called gave magnesia and carbonate of soda in frequently-repeated doses. Subsequently lime-water with breast-milk was administered; then nitrate of potash in combination with the compound tragacanth powder. The infant survived 25 days. Great praise is due to the skilful treatment of the surgeon in attendance, Mr. Tatham of Wandsworth. Perhaps it would have been better, if chalk had been administered in place of magnesia and soda, as the sulphuric acid forms soluble aperient salts with these bases; and the diarrhœa thereby induced might prove troublesome.

On *dissection,* no distinct morbid appearances were discoverable in any part of the alimentary tube.

The matter, that was at first rejected by vomiting, was of a glairy grumous nature, thick, and of an almost black colour. This was subsequently analyzed by Mr. Taylor, and shewn to contain distinct traces of sulphuric acid, by testing it with a salt of barytes, the white precipitate so formed being insoluble on the addition of nitric acid. With respect to the physical appearances of the vomited matter, Mr. Taylor observes that the acid "*must have been evidently taken in a concentrated form, from the dark and carbonized appearance of the matter ejected.** *It is only concentrated vitriol which thus chars and decomposes blood, mucus, and other organic substances;* and by this action it becomes itself decomposed and partially lost."

The report drawn up by Mr. Taylor was for the use of the Wandsworth magistrates. The person upon whom suspicion fell, was a servant girl in charge of the child. The sulphuric acid had been obtained for the preparation of blacking. That the infant had perished from the effects of this most corrosive poison, somehow given to it, could not be disputed, after the convincing medical and chemical evidence adduced; but, then it was argued by the prisoner's counsel, that the oil of vitriol had been administered, in mistake, for aniseed water, by the mother of the child, and not by the servant. The jury, being not satisfied of the guilt of the latter, acquitted her.

Mr. Taylor enters upon a lengthened examination of the various circumstances connected with the medical and chemical evidence of the case, to shew that it could not have been the mother who had accidentally administered the poison. The facts and reasonings which he adduces must satisfy, we should think, every reader.

2. The next case reported is one of Profuse Salivation, following the use of small doses of Calomel in Nephritis, and proving fatal. The case occurred about twelve months ago at Reading, and the party inculpated

* "I place these words in italics, as the defence set up was that the acid was taken in so *diluted* a state, as not to have the power of *carbonizing sugar!* See Mr. Tatham's evidence, p. 417."

was an unlicensed practitioner who resided in that town. It seems scarcely necessary to give the particulars; our chief object in mentioning the case being to allude to the point of practice as to the propriety of administering mercury, to the extent of salivation, in cases of disease of the kidneys. According to Mr. Taylor's observations, " it may be clearly admitted as a general rule that mercurial preparations, even in small doses, are liable to produce excessive salivation in persons affected with renal disease." Certain it is that the system is unusually apt, in many such cases, to be most rapidly and severely mercurialized. Mr. Harrison, a surgeon of Reading, mentioned, upon the present trial, two cases of this sort. One occurred in a woman affected with Bright's disease; one drachm of mercurial ointment was rubbed in upon her side; profuse salivation and exfoliation of the jaw followed. In the other case, said to be of Atrophy of the Kidneys, the most intense salivation was produced by two grains of the *Hydrarg. c̄ Cretâ!*

The following extract from a letter recently addressed by Dr. Christison to Mr. Taylor, on the point under consideration, will meet with attention: it takes a sober and, we think, a right view of the question.

" In cases of this disease (granular degeneration of the kidneys) I have repeatedly observed that mercurial action is brought on by unusually small doses of the compounds of mercury, or unusually soon; and I have also sometimes observed the action to be unusually violent in such circumstances. I cannot say that I have ever seen violent uncontrollable action induced on any occasion; neither have I seen mercurial action, even in its slighter degrees, brought on by the very small doses (such as a single dose, or two doses of calomel), which are well known to act with violence in certain constitutions not otherwise materially unsound.

" I suppose, from what some London physicians have written on the subject, and from the case alluded to by you in your letter, that the constitutional sensibility to mercurial action, induced by disease of the kidney, has been noticed in a greater degree in London than here. At all events, we are not deterred here, by any thing we have witnessed, from using mercury as an adjunct to other remedies, such as diuretics and cathartics, in granular disease of the kidneys. We watch it more narrowly, find its constitutional action more easily excited, but experience no difficulty in controlling this action when brought on." P. 458.

8. Case of Poisoning by Arsenic; Pregnancy not followed by Abortion; Detection of the Poison in an Entozoon.

A young unmarried woman died, after seven hours' illness, of what was at first supposed to be violent English Cholera. Suspicions, however, being awakened, the body was examined; and the surgeon in attendance reported that he had reason to believe that the deceased had died from the effects of some irritant poison: she was in the fifth month of pregnancy. On analysing the contents of the alimentary tube, a large portion of arsenic was readily discoverable.

As there was a *lumbricus* found in the ileum, Mr. Taylor was desirous of ascertaining whether it had become impregnated with the poison which had caused death. It was first carefully washed from all adhering mucus or blood, and then cut into pieces and boiled for two hours in one part of muriatic acid and eight of water. Traces of arsenic were readily discovered in the acid liquid, previously filtrated. Mr. Taylor expresses his regret that he had not the opportunity of examining the fœtus in this case, as

"the analysis of some of the organs might have thrown light on the absorption and diffusion of poisons."

4. Passing over three cases of Poisoning by Corrosive Sublimate, we come to the case of Poisoning by Lead, which is interesting alike in a diagnostic and in a medico-legal point of view. When the patient, a woman of the most intemperate habits, was received into Guy's Hospital, it was not known that she had swallowed any poisonous matter. Constipation, vomiting, and severe constant pain in the loins and abdomen were the most conspicuous symptoms of her case. On the third day after admission, *a distinct blue line was noticed on both gums;* and it was found at the same time that the patient could not extend her wrists completely out. She died, a few days subsequently, convulsed. It seems that no direct information could be obtained by the medical officers as to whether the woman had ever swallowed any poison, or not; but the symptoms now named naturally suggested the probability of lead having been the toxic agent. It was therefore an interesting point to determine whether the parenchymatous substance of any of the viscera contained traces of this metal.

The dissection, we may remark, revealed nothing very satisfactory to account for death; perhaps the most remarkable appearance was the irregularly contracted and distended state of the large intestines. We shall give Mr. Taylor's account of his chemical examination of the Liver—the organ, in which Orfila and others have generally best succeeded in detecting the presence of mineral poisons in the tissues of the body.

"The liver was dried and incinerated, and the ash thus obtained was digested in water, containing one-eighth part of strong nitric acid. The acid solution thus obtained contained a large quantity of phosphate of lime and iron, and left, on evaporation, silica, probably derived from the crucible. The acid liquid, evaporated to dryness, was again digested in a small quantity of very diluted nitric acid and filtered. Diluted sulphuric acid gave, with this liquid, a white precipitate, not entirely soluble in potash, because phosphate of lime was precipitated by the alkali from the acid solution. The acid liquid was also precipitated, of a deep greenish-black, by a current of sulphuretted hydrogen gas; and on adding more nitric acid, the sulphuret of iron was removed, and a light-brown precipitate remained, which was sulphuret of lead. A portion of the original liquid was then strongly acidified with nitric acid: sulphuretted hydrogen was passed into it, and a brown precipitate of sulphuret of lead only was now thrown down, the iron being suspended. A portion of the original liquid, nearly neutralized by potash, gave, in a few seconds, the brilliant yellow precipitate, in crystalline scales, of iodide of lead. The galvanic test of zinc and platina did not answer, the quantity of lead present being too small. The tests acted clearly and decidedly, leaving no doubt that lead was present in the liver of the female in comparatively large quantity." P. 473.

The case which follows is one, wherein a middle-aged woman swallowed an ounce and a half of sugar of lead with the intention of committing suicide. Fortunately she vomited at the time, and no alarming symptoms ensued.

The concluding cases of Poisoning, one from a very large dose of Oil of Bitter Almonds, and the other from Prussic Acid, are instructive in several points of view. In both instances, a period of several minutes elapsed after the patients had taken the poisonous dose, before the fatal effect was induced. In the *first*, it is supposed that the man swallowed nearly five or six drachms of the oil of bitter almonds; and that he retained for four or five minutes not only his full consciousness, so as to reply rationally to questions addressed to him, but even his ability to get up from his chair and walk towards the door of the apartment. Then the symptoms of poisoning appear to have come on suddenly; first vomiting, and almost immediately afterwards insensibility, convulsive breathing, slight opisthotonos, and death. There were no convulsions, if we except the partial opisthotonos; nor was there any scream or shriek preceding death. This case therefore, as well as others, clearly shews that neither of the symptoms now named can be regarded as, at all, of uniform occurrence in this kind of poisoning.

In the *second* case, two drachms, it is supposed, of the dilute Hydrocyanic Acid of the Pharmacopœia were taken. "The peculiar interest of this case results from the duration of the power of volition after so large a dose had been taken, the deceased having descended *thirty stairs*, and walked about *twenty paces*, before he became powerless; this happened whilst he was in the act of attempting to open the street-door. The proprietor of the house and the servant were in a back room on the ground-floor, level with the coffee-room he passed through upon coming down stairs, and they saw him proceed directly to the door: the servant advanced to open it for him, and had no sooner gained his side than he fell, and, to use her own words, 'threw his arms about, and made a noise in breathing, fetching it hard, but there was nothing approaching to a scream:' he very soon became still."*

Mr. Taylor makes the following remarks upon this case:—

"Thus, then, an individual was here enabled to walk, and otherwise exert his bodily powers, after having swallowed more than *two grains and a half* of anhydrous prussic acid! Many facts of this kind have been recorded of late years; but I am not aware of any instance in which such a series of voluntary acts has been performed, and such a power exerted, after so large a dose of the poison had been taken. This case, therefore, creates an additional caution; namely, that we must make full allowance for the occurrence of some delay in the accession of symptoms, even where the dose is large." P. 492.

In this instance also there was no *scream* before death. It may be also worthy of notice, that the medical gentleman, Mr. Lowe of Aldersgate Street, who was called to the deceased, could perceive no odour of prussic acid about his mouth, although a considerable dose had been taken only a few minutes before.

In closing our notice of these painfully-instructive reports, we cannot but again express our astonishment that the most active poisons should be so readily procurable, in every part of the country, by persons who can have no proper object in view in purchasing them. It is high time that chemists and others, who deal in such dangerous articles, should be prohibited by penal enactments from pandering—we can call it nothing else—to the awful crimes of Suicide and Murder.

---

* This description of the symptoms in this instance by an eye-witness closely corresponds with the account given by Tawell of those, which he witnessed in the case of Sarah Hart.

